# High-throughput segmentation of unmyelinated axons by deep learning

**DOI:** 10.1038/s41598-022-04854-3

**Published:** 2022-01-24

**Authors:** Emanuele Plebani, Natalia P. Biscola, Leif A. Havton, Bartek Rajwa, Abida Sanjana Shemonti, Deborah Jaffey, Terry Powley, Janet R. Keast, Kun-Han Lu, M. Murat Dundar

**Affiliations:** 1 Department of Computer and Information Sciences, Indiana University, Purdue University, Indianapolis, IN 46202 USA; 2 Department of Neurology, Icahn School of Medicine at Mount Sinai, New York, NY 10029 USA; 3 Department of Neuroscience, Icahn School of Medicine at Mount Sinai, New York, NY 10029 USA; 4 James J. Peters Department of Veterans Affairs Medical Center, Bronx, NY 10468 USA; 5 Bindley Bioscience Center, Purdue University, West Lafayette, IN 47906 USA; 6 Department of Computer Science, Purdue University, West Lafayette, IN 47906 USA; 7 Department of Psychological Sciences, Purdue University, West Lafayette, IN 47907 USA; 8 Department of Anatomy and Physiology, The University of Melbourne, Melbourne, VIC 3010 Australia; 9 Weldon School of Biomedical Engineering, Purdue University, West Lafayette, IN 47907 USA

**Keywords:** Neuroscience, Neurology, Computational neuroscience, Image processing, Machine learning

## Abstract

Axonal characterizations of connectomes in healthy and disease phenotypes are surprisingly incomplete and biased because unmyelinated axons, the most prevalent type of fibers in the nervous system, have largely been ignored as their quantitative assessment quickly becomes unmanageable as the number of axons increases. Herein, we introduce the first prototype of a high-throughput processing pipeline for automated segmentation of unmyelinated fibers. Our team has used transmission electron microscopy images of vagus and pelvic nerves in rats. All unmyelinated axons in these images are individually annotated and used as labeled data to train and validate a deep instance segmentation network. We investigate the effect of different training strategies on the overall segmentation accuracy of the network. We extensively validate the segmentation algorithm as a stand-alone segmentation tool as well as in an expert-in-the-loop hybrid segmentation setting with preliminary, albeit remarkably encouraging results. Our algorithm achieves an instance-level $F_1$ score of between 0.7 and 0.9 on various test images in the stand-alone mode and reduces expert annotation labor by 80% in the hybrid setting. We hope that this new high-throughput segmentation pipeline will enable quick and accurate characterization of unmyelinated fibers at scale and become instrumental in significantly advancing our understanding of connectomes in both the peripheral and the central nervous systems.

## Introduction

Recent neuroimaging technologies have advanced the knowledge of the nervous system, but primarily at the polar extremes—detailed nano-scale reconstructions including every synapse on one hand and global circuit projections on the other. With the growing interest in connectomes, there are now, for example, several elegant, complex, and nearly complete 3D structural reconstructions of small blocks of the cortex. As impressive as such microconnectomic reconstructions are, they are not high-throughput and do not cover the whole nervous system. A proof of principle prepared by Motta et al.^1^ is representative. Reporting on their detailed digital reconstruction of a single small block of layer 4 in mouse somatosensory cortex, the investigators admitted that the model, albeit computer-aided, required 4000 human work-hours to construct. Such reconstructions can certainly be informative, but they are unlikely to be used routinely on a high-throughput basis in the foreseeable future. Furthermore, they may fail to identify axonopathies—it is noteworthy that the tissue sample’s axonal input and output connections were not characterized in the Motta et al. reconstruction. Additionally, they have not been validated, and the particular image analysis may not generalize to non-cortical (or even other cortical) sites.

As Kasthuri et al.^2^ argue in their “projectome” paper, “some applications, such as determining the organization of the neural projections in the brain, are better served by comprehensive imaging of very large samples at lower resolution.” Since many neurological diseases (e.g., diabetes, parkinsonism, multiple sclerosis, and alcoholism) are known to involve axonopathies that affect the projectome and not just sites in a connectome block of tissue, the field cannot ignore the billions of axons that supply the trillions of synapses. We lack the complementary tools to evaluate projectomes as well as limited tissue blocks. Connectomics cannot be studied realistically without sound axonal information.

### Current state of the art

#### Axon segmentation

Crucially, for understanding connectomes, no automated axon analyses yet evaluate, let alone validate, their assessments of the most prevalent axons in the nervous system, the unmyelinated fibers. The advances in connectomics have not yet been matched by advances in axon segmentation^1,3^. For example, Kasthuri et al.^3^ found a small minority of myelinated axons and a vast majority of unmyelinated fibers in their connectomic analysis of the mouse neocortex. Yet, available software packages do not segment and analyze unmyelinated fibers. Studies of connectomes and “projectomes” are forcibly skewed toward myelinated assessments. The search for axonopathies in non-traumatic developmental and mental disorders (autism, ADD, schizophrenia, dyslexia, etc., that may well be unmyelinated fiber pathologies) is being forced to commit the fallacy of “looking under the lamppost.”

Traditionally, the images employed as input for the downstream spatial analysis or qualitative descriptive analysis in neuroanatomy have been processed manually or semi-manually. The manual techniques involve the use of a computerized planimetry with a digitizing tablet^4^. The semi-manual approach proceeds through multiple grayscale image processing operations involving global binarization followed by a complex set of semantic rules applied to axon shape, size, and morphology to remove false positives and clean images^5^. An improvement in the semi-manual techniques utilized locally adaptive thresholding and an extensive application of morphological operations in post-processing^6,7^. The traditional analysis produces outputs compatible with spatial statistical methods, including myelinated fiber position, diameter, shape, and myelin thickness.

It is important to emphasize that almost all the attention guiding the development of (semi-)automated approaches has been focused on myelinated fibers. The myelin provides a source of contrast for transmission electron microscope (TEM) images and consequently is of critical importance to segmentation approaches based on the intensity thresholds found in greyscale values. If the unmyelinated fibers are to be counted, the semi-automated threshold methods assume the manual processing mode^6^. The more modern semi-automated or fully automated artificial intelligence (AI)-based solutions have been produced and improved with a similar mindset. Consequently, the widely accessible research segmentation software or non-commercial implementations of research algorithms custom-developed in neuroscience laboratories continue their focus on myelinated fibers for scientific and practical reasons^8–10^. The experience of other research groups experimenting with machine learning (ML) and deep-learning approaches in neuroimaging demonstrates that, as before, all the work in ML-driven segmentation has been tested within the context of the much easier problem of myelinated fibers^11–13^.

#### Cell and nucleus segmentation

Segmentation of biological objects (cells, nuclei, other organelles) has long been considered as one of the challenging automation tasks in microscopic image analysis. Traditionally, it has been tackled by the watershed algorithm^14,15^, which creates a topological map of the image where basins represent structures to be segmented, or by region growing^16^, where seed pixels are expanded to fill uniform areas. However, the watershed algorithm is prone to over-segmentation, especially in noisy images as basins are created from local minima, and under-segmentation in low-contrast images because vague boundaries between cells may cause multiple structures to blend. While a merging step^17^ may mitigate over-segmentation, it often requires the application of heuristic rules describing sizes or shapes of the structures to remove spurious detections. In general, the segmentation pipelines based on mathematical morphology generalize poorly. Tuned correctly, they may work with specific imaging modalities but fail to perform well when employed with different sensors, resolutions, or contrast levels. Importantly, they do not make use of contextual information, e.g., about the typical cell shape, size, and orientation. The flood-filling networks partially mitigate these shortcomings^18^ by learning the features with deep architecture and incorporating contextual information with a recurrent neural network.

Recent segmentation methods relying on deep convolutional neural networks have demonstrated significant progress^19–22^: among these U-Net^19^, which uses a U-shaped encoder-decoder network architecture, has become the most prevalent network architecture for segmentation problems in biomedical images. However, similarly to the legacy techniques, deep learning algorithms are highly tuned and optimized for specialized applications, and they generalize poorly or fail when applied to related tasks. In an effort to improve the usability of networks for cell segmentation, “generalist” variants have been proposed. For instance, Falk et al. configure U-Net hoping to achieve a generalized deep-learning solution for various cell detection and segmentation jobs^23^. *CellPose*^24^ uses a U-Net architecture to learn the mapping between cell masks and gradient vector fields in images using annotated cell data from a wide range of microscopic applications as well as nonmicroscopic data comprising repeated blob-like structures. Yet, as shown in Fig. 11c *CellPose* does not perform well segmenting unmyelinated fibers. This unsatisfactory outcome is caused by the fact that EM images possess not one but multiple types of blob-like structures (Schwann cell nuclei, blood vessels, myelinated and unmyelinated fibers) with significant differences in size, shape, and contrast. A “generalist” model such as *CellPose* is unable to discriminate among structures with different characteristics. The lack of readily available solutions for our neuroanatomical studies led us to develop a more specialized application optimized for unmyelinated fiber segmentation.

#### Challenges in unmyelinated fiber segmentation

Compared to myelinated fibers, which are generally characterized in TEM images by a bright region (the axon) surrounded by a dark ring (the myelin sheath) of relatively uniform thickness, unmyelinated fibers (UMFs) exhibit considerable variability in appearance between and within images. A general UMF segmentation algorithm must thus handle a wide range of inputs and use contextual clues to differentiate fibers with ambiguous appearance.

**Figure 1:**
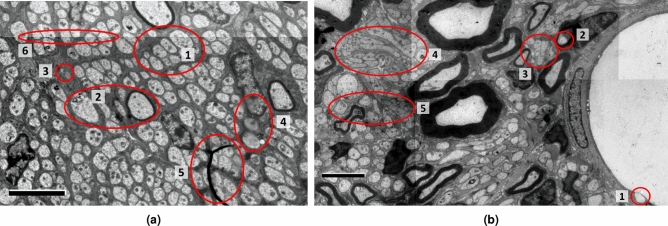
Variability of unmyelinated fibers (UMFs). (**a**) UMFs of different size (**1**), myelinated fibers with similar size and shape (**2**), fascicle texture mimicking UMFs (**3**), imaging artifacts (**4**, **5**) and different contrast between tiles (**6**). (**b**) Vescicles in the blood vessel (**1**) and in a myelinated fiber (**2**) mimicking UMFs, clumped UMFs (**3**) and UMFs with different shape (**4**) or contrast (**5**). Scale bars: 2 $\upmu$m.

**Figure 2:**
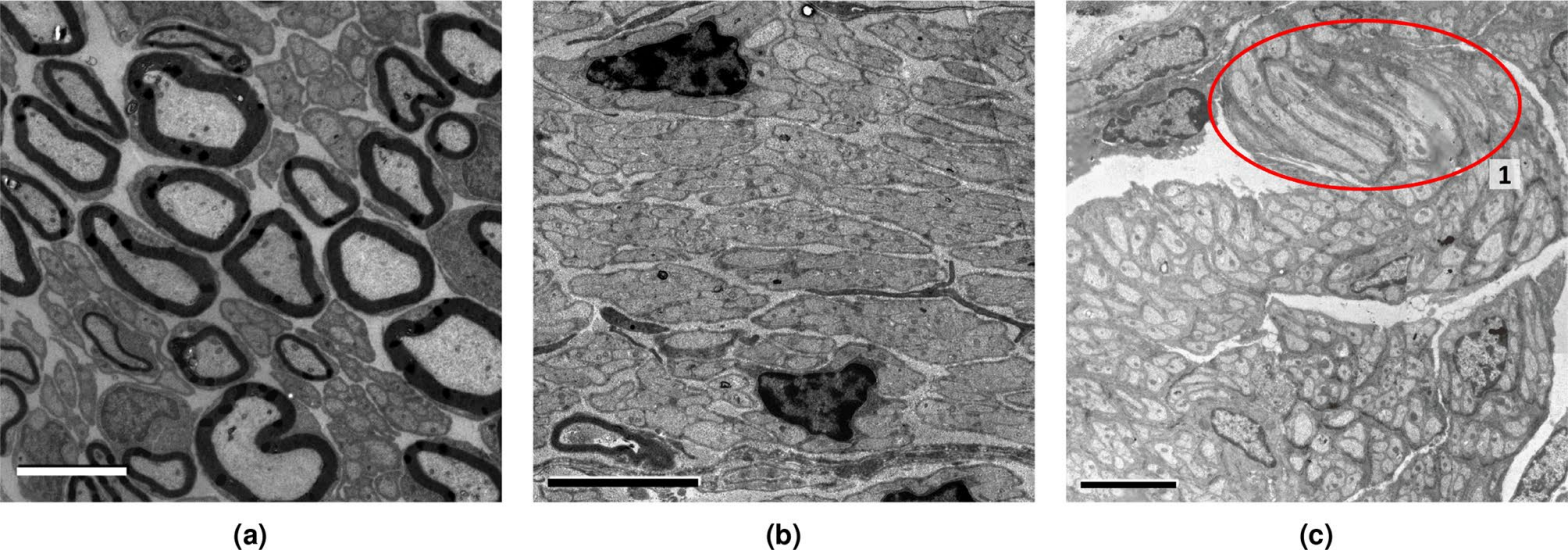
Images with different characteristics. (**a**) Myelin-rich regions with dark unmyelinated fibers (UMFs). (**b**) Low-contrast fibers. (**c**) Light UMFs in myelin-poor regions with a Remak bundle highlighted in (**1**). Scale bars: 4 $\upmu$m.

**Figure 3:**
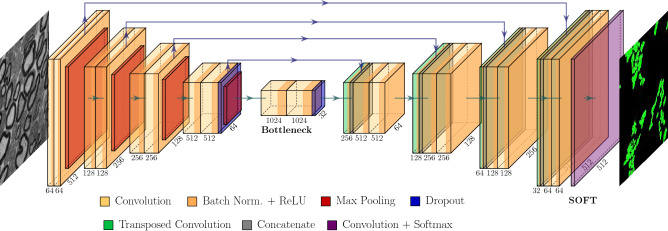
U-Net segmentation model on tiles of size $512 \times 512$ pixels, with 4 down-sampling (encoder) and up-sampling (decoder) blocks linked by skip connections. A batch normalization layer is inserted before the ReLU non-linearity in all the convolutional layers and dropout adds further regularization in the bottleneck. The image is drawn by PlotNeuralNet V1.0.0^27^.

**Figure 4:**
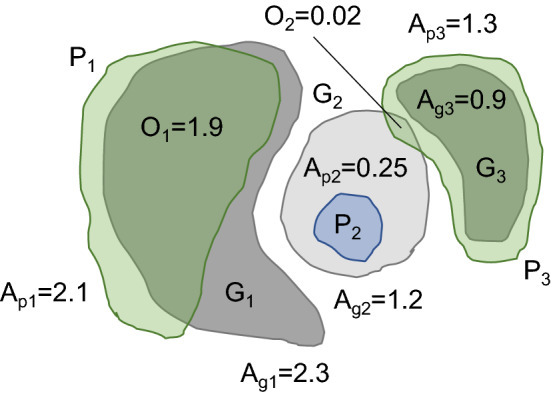
Illustrative example of *SQ* and *RQ* computations, with predictions in green (true positive) or blue (false positive) and annotations in solid gray (true positive) or light gray (false negative).

As in the case of myelinated fibers, UMFs vary significantly in size, even in the same image region (Fig. 1a, 1). UMFs tend to have a circular shape because the images represent cross-sections. Still, some fibers may have elongated elliptical shapes (Fig. 2b), elongated shapes with lobes (Fig. 1b, 4) or sickle shapes and are often aggregated in Remak bundles (Fig. 2c, 1). UMFs with different shapes may be clustered in separate, uniform regions (Fig. 2b, c) or intermingled (Fig. 1b, 4). The fibers are often clumped into islands with only thin separating borders (Fig. 1b, 3), and segmentation algorithms often merge them.

TEM images of nerve cross-sections also show significant variability not only within component categories (i.e., UMFs, myelinated fibers, and Schwann cell nuclei) but also in their overall distribution and frequency. A primary distinction can be noted between images rich in myelinated fibers (such as Fig. 2a) and images almost devoid of them, with only a few Schwann cell nuclei (Fig. 2b). UMFs will appear mostly circular when the sample is cut perpendicularly to the fiber direction (Fig. 1a). Still, some images may have regions of elongated objects owing to the sample preparation or because the fibers branch out of the nerves at certain angles (Fig. 2c). Moreover, UMFs may have a distinctive appearance caused by the automatic exposure setting during TEM imaging. Here we show three examples: dark fibers on a bright background (Fig. 2a), low-contrast fibers (Fig. 2b) and light fibers on a dark background (Fig. 2c). Several blob-like structures may mimic the features of UMFs, increasing the difficulty of the task. A non-exhaustive list includes myelinated fibers with similar shape and size (Fig. 1a, 2), blob-like features in the fascicle (Fig. 1a, 3), near blood vessels (Fig. 1b, 1), or in myelinated fibers (Fig. 1b, 2).

**Figure 5:**
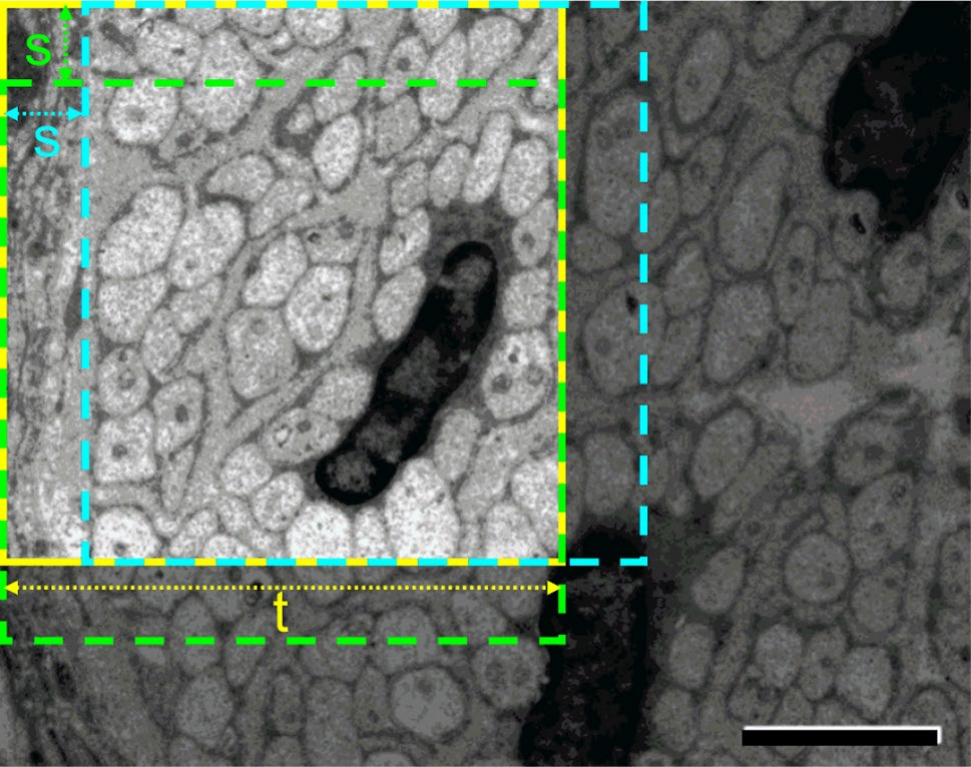
Inference for an image is performed by processing overlapping tiles. The first tile of size *t* is shown in solid yellow, the next horizontal tile in dashed cyan and the next vertical tile in green. A stride of *s* has been applied. After the entire image is processed majority voting is applied to pixel-wise detections. Scale bar: 2 $\upmu$m.

**Figure 6:**
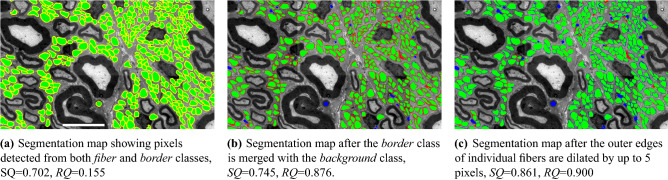
Illustration of the post-processing. Green is used for pixels assigned to the fiber class, yellow for those assigned to the border class, red false negatives, and blue false positives. Scale bar: 6 $\upmu$m.

The imaging process adds further variability. For instance, the images in our dataset were acquired with resolutions of 11.9 nm/pixel and 13.7 nm/pixel, and different contrast depths. All the images are mosaics of several partially overlapping tiles, and thus they may show varying intensity levels on the seams or stitching misalignment artifacts (Fig. 1a, 6). Some regions may be smeared or blurred (Fig. 1a, 4) or have foreign objects in the foreground partially covering the axons (Fig. 1a, 5).

Handling different cases requires (a) a careful selection of training images covering main variability factors and (b) a model with enough capacity and expressiveness to learn them. To address (a), we train the model on images of different sizes and resolutions acquired from multiple areas of the samples representing several animals of both sexes. Contrast differences are handled by equalizing histograms of the regions passed to the model (see section “Training parameters”). Large and elongated fibers appear with lower frequency compared to small, round fibers, and thus the sampling strategy, discussed in section “Tile sampling” is essential to ensure balanced coverage. To address (b), we test U-Net networks with different depths, loss functions and tile sizes, and select the best performing model for final training (see section “Training parameters”). A dataset of TEM images with annotated unmyelinated fibers is not publicly available, and thus another contribution of this work is the creation of a large dataset of composite images of nerve cross-sections (see sections “Sample collection” and “Training parameters”).

## Methods

### Sample collection

Two types of samples were used to develop and test the model. Vagal nerve samples were prepared at Purdue University, while pelvic nerve samples were obtained at the University of Melbourne. All procedures involving animals were performed according to ARRIVE guidelines^25^. The sample collection and processing protocols are described in detail below.

#### Vagal nerve samples

Sprague–Dawley rats (2–4 months old; n = 2 each, male and female; RRID:RGD_737903; Envigo, Indianapolis, IN) were housed in shoe-box cages with bedding material in an Association for Assessment and Accreditation of Laboratory Animal Care-approved colony room, temperature (22–24 °C) and humidity (40–60%) controlled. The room was maintained on a 12:12 h light–dark schedule. Pelleted chow (2018 Teklad global 18% protein rodent diet; Envigo, Indianapolis, IN, USA) and filtered tap water were provided ad libitum. All husbandry practices conformed to the National Institutes of Health (NIH) Guide for the Care and Use of Laboratory Animals (8th edition) and were reviewed and approved by the Purdue University Animal Care and Use Committee. All efforts were made to minimize any suffering as well as the number of animals used. Animals were perfused according to the following procedure. Specifically, they were overdosed with anesthetic (intraperitoneal injection of ketamine/xylazine (Patterson Veterinary Supply, Devens, MA/Akorn Animal Health, Lake Forest, IL, USA), 275 mg/kg of ketamine/27.5 mg/kg of xylazine), exsanguinated with fresh physiological saline, and then perfused with a fresh solution of EM-grade fixatives (2% paraformaldehyde/1.25% glutaraldehyde (Fisher Scientific, Hampton NH/Electron Microscopy Sciences, Hatfield, PA) in 0.1 M phosphate-buffered saline [PBS], pH = 7.3) for 20 min. Nerve bundles, trunks, and branches were immediately dissected out and moved into the same fixatives overnight at 4 °C on an oscillating platform. The following morning, the tissue specimens were rinsed ($3 \times 30$ min at 4 °C) in PBS (0.1 M, pH = 7.3), transferred to individual shipping vials under PBS, and shipped overnight to the TEM laboratory for further processing and microscopy.

#### Pelvic nerve samples

Pelvic nerve dissection followed procedures approved by the Animal Ethics Committee of the University of Melbourne and in compliance with the Australian Code for the Care and Use of Animals for Scientific Purposes (National Health and Medical Research Council of Australia). Two male Sprague-Dawley rats (7–8 weeks old) were sourced from the Biomedical Sciences Animal Facility (University of Melbourne), housed under a 12-h light-dark cycle, in a temperature-controlled room with ad libitum access to food and water. Under anesthesia (100 mg/kg ketamine, 10 mg xylazine i.p. (Lyppard, Keysborough, Australia)) animals were perfused transcardially with saline (0.9% sodium chloride containing 1% sodium nitrite and 5000 IU/ml heparin (Ellar Laboratories, Tullamarine, Australia)), followed by fixative (2% paraformaldehyde and 1.25% glutaraldehyde (Proscitech, Thuringowa, Australia)) in 0.1 M PBS, pH 7.3) for 15–20 min. The detailed perfusion procedure is described in^26^. Each pelvic ganglion with its attached pelvic nerve was then dissected, postfixed in the same fixative for 18–24 h at 4 °C, washed in PBS ($3 \times 30$ min), stored in PBS, and couriered to the TEM laboratory for further processing and microscopy.

### Sample processing and imaging

At the TEM laboratory the tissues were rinsed in PBS and fixed in 1% osmium ($\mathrm {OsO_4}$) solution. The tissues were then dehydrated in a series of ethanol and 100% propylene oxide, and embedded in Epon plastic resin. Cross sections of embedded samples were obtained (0.5 $\upmu$m), and stained with a 1% toluidine blue solution for light microscopic (LM) analysis under a Nikon Eclipse E600 microscope. This step was to assure that the fascicles are complete with intact perineurium and endoneurium space before moving forward the tissue processing to TEM instrument. Ultrathin sections (70–90 nm) were collected on single-hole formvar-coated copper grids and counterstained with uranyl acetate and lead citrate. The samples were analyzed using a Tecnai G2 Spirit TEM (FEI, ThermoFisher Scientific), and the full cross section for each nerve was captured using a Gatan Orius SC 1000B digital camera (Gatan, Inc.) at 3200$\times$ magnification.

For the purpose of segmentation, followed by quantification of the spatial arrangement of the axons (see section “Validation of spatial distribution of unmyelinated fibers”), the individual TEM tiles need to be stitched together to provide a single ultra-large TEM image (typically 20–200 tiles). The TEM tiles were assembled using either Adobe Photoshop (Adobe Inc., San Jose, CA, USA) or Image Composite Editor (Microsoft Corp., Redmond, WA, USA). In both cases, specifically, the *Auto* merge option was used in which the software automatically analyzed all TEM tiles and applied an appropriate transformation to best stitch the tiles. Prior to merging, images were manually adjusted to minimize contrast variation between tiles. Manual segmentation of unmyelinated axons was performed with Neurolucida 360 version 2020.3.3 (MBF Bioscience, Williston, VT USA). The complete list of manually segmented images is in Table 1. Thin contours were manually drawn using the continuous tracing option in Neurolucida. The border thickness was manually adjusted to overlay the axon plasma membrane, but not to obscure adjacent tissues or the underlying fiber, and it ranges between 0.01 and 0.03 $\upmu$m.

**Table 1:** Training, validation, testing, and evaluation images available in our data repository.

Image ID	Image size	Split	# Annotated axons	Resolution (nm/px)	Nerve	Location	Sex
1	20372 $\times$ 27269	Train	13284	11.9	Vagus	Left cervical trunk	F
2	7953 $\times$ 5781	Train	1533	11.9	Vagus	Ventral gastric branch	F
3	8446 $\times$ 7258	Train	4476	13.7	Vagus	Ventral gastric branch	M
4	4128 $\times$ 4068	Train	1029	13.7	Vagus	Ventral gastric branch	M
5	5521 $\times$ 4971	Train	1894	13.7	Vagus	Ventral gastric branch	M
6	5262 $\times$ 7111	Train	9636	13.7	Vagus	Ventral abdominal trunk	M
7	8633 $\times$ 8866	Train	1507	11.9	Pelvic	$\le$2 mm from pelvic ganglion	M
8	3891 $\times$ 3334	Train	252	11.9	Pelvic	$\le$2 mm from pelvic ganglion	M
9	2754 $\times$ 2958	Train	231	11.9	Pelvic	$\le$2 mm from pelvic ganglion	M
10	3357 $\times$ 3823	Train	271	11.9	Pelvic	$\le$2 mm from pelvic ganglion	M
11	4419 $\times$ 5701	Train	483	11.9	Pelvic	$\le$2 mm from pelvic ganglion	M
12	5064 $\times$ 7207	Train	595	11.9	Pelvic	$\le$2 mm from pelvic ganglion	M
13	5869 $\times$ 6268	Train	992	11.9	Pelvic	$\le$2 mm from pelvic ganglion	M
14	4028 $\times$ 3513	Train	445	11.9	Pelvic	$\le$2 mm from pelvic ganglion	M
15	7941 $\times$ 6372	Train	1333	11.9	Pelvic	$\le$2 mm from pelvic ganglion	M
16	11129 $\times$ 7962	Train	2418	11.9	Pelvic	$\le$2 mm from pelvic ganglion	M
17	2004 $\times$ 1336	Validation	19	11.9	Pelvic	$\le$2 mm from pelvic ganglion	M
18	2804 $\times$ 4221	Validation	353	11.9	Pelvic	$\le$2 mm from pelvic ganglion	M
19	1558 $\times$ 1697	Test	364	11.9	Vagus	Ventral gastric branch	F
20	24746 $\times$ 20682	Test	12250	11.9	Vagus	Right cervical trunk	F
21	9935 $\times$ 8870	Evaluation	4379	13.7	Vagus	Ventral gastric branch	M

All images are obtained from rats. Training images are used for model training. Validation images are used to optimize model hyperparameters and other training options. Test images are used to evaluate stand-alone segmentation performance. Evaluation image is used to evaluate the algorithm in the expert-in-the-loop setting.

### Segmentation model

Our segmentation model (in Fig. 3) is a U-Net with 4 stages; all the convolutional layers have a batch normalization layer followed by a ReLU activation layer, and the bottleneck stage has additional dropout layers between convolutions. The model predicts three classes: unmyelinated fibers (*fiber*), background, and a boundary region between the previous two defined by the outer edge of each fiber (*border*). The role of the border class is to penalize errors between spatially adjacent fibers to more accurately segment individual instances of fibers. We divide the image into tiles since running the model on the whole extended field-of-view TEM images would require a prohibitively large amount of memory, whereas down-sampling the images would lead to loss of details. We tested multiple sampling strategies for training (see section “Tile sampling”). During testing, we sample tiles at regular intervals (see section “Final training, inference, and post processing”). The intensity histograms of the tiles were equalized before running the model to reduce local contrast differences.

Our algorithms were implemented in Matlab R2021a (Mathworks, Natick, MA, USA) using the Deep Learning and Image Processing toolboxes. Most of the experiments were performed on a Windows 10 machine equipped with a i9 9900 CPU at 3.1 GHz and an NVIDIA GeForce RTX 2080 GPU. We switched to a more advanced hardware configuration that includes an NVIDIA V100 GPU when the tile batches required larger memory.

### Training parameters

To reduce computational time, evaluation of different training configurations is performed with a subset of the dataset restricted to the pelvic nerve images in Table 1. More specifically, images 7–16 were used for training and images 17 and 18 were sequestered for validation. Around fifteen thousand tiles are extracted with a maximum of two thousand tiles per image. The training is run for four epochs for a total of thirty thousand iterations using stochastic gradient descent (SGD) with momentum on mini-batches of size two. The learning rate is set to an initial value of 0.01, and a decay rate of 0.1 is applied after each epoch.

If a modified sampling strategy is used (see section “Tile sampling”) the sampling parameters are adjusted to generate a dataset of the same size. The evaluation measures (section “Evaluation measure”) are computed on the tiles generated from validation images after applying the post-processing steps discussed in section “Final training, inference, and post processing”.

We selected training settings through an extensive search of hyperparameters, reported in Table 2. The default model is a 4-stage U-Net on tiles of 512 pixels, trained with a weighted cross-entropy loss and on tiles sampled based on area and circularity, extracting around 1000 tiles per image. Each hyperparameter is changed in isolation starting from this default model. In the following sections, we discuss each choice separately. First, we present our evaluation measure.

#### Evaluation measure

When manual annotations are available for an image, the performance of the automated segmentation is evaluated based on the *Panoptic Quality (PQ)* score^28^, a recently introduced evaluation measure for instance segmentation. Each connected component in the segmentation map is treated as a separate instance. *PQ* pairs annotated and predicted instances, restricting matches to an Intersection over Union (*IoU*) score of 0.5 or more because a uniqueness theorem^28^ ensures that each annotated region is paired to at most one predicted region and vice-versa. This, in turn, ensures that a greedy matching will give the same results as more expensive optimal matching approaches, such as the Kuhn-Munkres algorithm^29^. *PQ* is defined as a product of two other scores:

1 $$\begin{aligned} PQ = \underbrace{\frac{{\sum \nolimits _{(p,g) \in TP} {IoU} (p,g)}}{{|TP|}}}_{{\text {segmentation quality (}}SQ{\text {)}}} \times \underbrace{\frac{{|TP|}}{{|TP| + \frac{1}{2}|FP| + \frac{1}{2}|FN|}}}_{{\text {recognition quality (}}RQ{\text {) }}} \end{aligned}$$

where true positive (*TP*) denotes an instance detected by automated segmentation and matching an instance in the manual segmentation with an intersection-over-union (*IoU*) score of 0.5, or higher, false positive (*FP*) denotes an instance detected by automated segmentation but that do not match an instance in the manual segmentation with an *IoU* score of 0.5 or higher, false negative (*FN*) denotes an instance in the manual segmentation but not detected by automated segmentation with an *IoU* score of 0.5 or higher. The Segmentation Quality (*SQ*) score is defined as the average *IoU* score computed with all *TP* detections. The Recognition Quality (*RQ*) score is the $F_1$ score computed at the instance level. *SQ* measures how well the automated segmentation recovers annotated instances, while *RQ* measures instance-level detection performance, which is sensitive to merged fibers. In Table 2 we report both *SQ* and *RQ* scores under different settings.

**Table 2:** Evaluation results for different choices of hyper-parameters, measured in terms of *Segmentation Quality (SQ)* and *Recognition Quality (RQ)*, with training and inference times.

	SQ	RQ	Training (min.)	Inference (s)
**Network depth**				
5	0.753	0.778	346	187
4	0.757	0.816	269	152
3	0.781	0.616	219	119
2	0.758	0.214 176	89	
**Loss**				
Weighted CE	0.757	0.816	269	152
Generalized dice	0.324	0.184	276	151
Focal	0.756	0.619	262	151
No border class	0.318	0.150	262	150
**Tile size**				
256	0.769	0.647	79	250
384	0.767	0.756	169	216
512	0.757	0.816	269	152
524 without padding	0.766	0.729	227	4.5
768	0.769	0.733	332$^{\dagger }$	54$^{\dagger }$
**Tile sampling**				
Area-based	0.757	0.816	269	152
Random	0.759	0.742	270	150
Fiber-centered	0.786	0.663	213	148
Proportional	0.783	0.675	213	150

Inference times are measured on image 18. CE denotes cross-entropy.

$$^{\dagger }$$Using a V100 GPU.

Figure 4 uses a toy example to illustrate the computation of *SQ* and *RQ*. Given predicted instances $P_i$ and annotated instances $G_j$ with instance areas $A_k$ and overlap areas $O_k$, we first compute the *IoU* scores $o_{ij}$ between all matching pairs. The only non-zero values are $o_{11}=1.9/(2.3+2.1-1.9)=0.76$, $o_{22}=0.25/1.2=0.21$, $o_{33}=0.9/1.3=0.692$ and $o_{32}=0.02/(1.3+1.2-0.02)=0.008$. Only $o_{11}$ and $o_{33}$ have *IoU* larger than 0.5, thus $P_1$ and $P_3$ are considered true positives, $P_2$ is considered a false positive and $G_2$ is considered a false negative. As we have only two true positives, one false positive and false negative *SQ* and *RQ* are computed as follows $SQ=(0.76+0.692)/2=0.726$ and $RQ=2/(2+0.5+0.5)=0.66$.

It is important to note that *IoU*, true positives, false positives, and false negatives are computed *per instance* and not per pixel; the per-pixel IoU is known as the Jaccard score, and the per-pixel $F_1$ score is known as the Dice score in the literature, and they both ignore correspondences between individual instances. The correct separation of fibers is critical for downstream processing of the detected objects (see section “Validation of spatial distribution of unmyelinated fibers”), but a segmentation algorithm may still achieve an excellent per-pixel score while ignoring the small areas between regions and thus missing individual instances. Therefore, unlike earlier studies that use pixel-wise evaluation, we strongly support the use of per-instance *SQ* and *RQ* scores for segmentation.

#### Tile size

The default setting for tile size is chosen as $512 \times 512$ pixels. A larger tile includes more context and thus may improve performance, but it also increases the amount of memory and processing time required to train and execute the model. On the other hand, a smaller tile size requires a larger number of tiles to evaluate to cover a given image, slowing down inference. We see some degradation in *RQ* for tiles of size 384 and an even more considerable decrease for 768. The default setting uses convolution with padding. Convolutions without padding are proposed in the original U-Net paper^19^ to remove border effects due to the application of filters on regions outside the input tile. We tested a tile size of 524 using convolutions without padding, leading to output size of 340 pixels. However, this arrangement resulted in worse outcomes and is not employed in the final model.

#### Loss function

The default setting of the loss function uses a per-pixel *weighted cross-entropy loss* with class weights that are inversely proportional to the frequency of the class in the training tiles. The border class has the largest weight due to its small footprint, and it plays the role of a border loss term, strongly penalizing segmentation errors between spatially adjacent or contiguous axons. This, in turn, is important in correctly separating different fiber instances. As shown in Table 2 removing the border class while keeping the weights reduces accuracy significantly, a direct result of numerous fibers being merged in the segmentation map.

We also considered two other loss functions: generalized dice loss and focal loss. The *generalized dice loss*^30^ is a per-pixel $F_1$ score, computed separately for each class, weighted by inverse class frequency and averaged. Despite being sensitive to over-segmentation (false positives) and under-segmentation (false negatives), the accuracy is low compared to the default setting. The *focal loss*^31^ weights the cross-entropy loss by a term $(1-p_c)^\gamma$, where $p_c$ is the predicted probability of the correct class *c* and $\gamma =0.25$ is a tuning parameter, boosting the weights of pixels classified with low prediction probability in the overall loss. Even without explicit class weighting, the border class is automatically boosted as it is harder to fit and the results are similar to the class-weighted cross-entropy.

#### Tile sampling

Sampling tiles randomly or on a lattice is inefficient, because a large number of tiles outside the fascicle do not have fibers, and thus do not give information on how to discriminate fibers from background. The random tile sampling strategy lowers the *RQ* score as seen in Table 2. Moreover, sampling the same number of tiles for each image gives better results than sampling a number of tiles proportional to the image size, emphasizing the importance of covering a diverse set of tiles for robust deep learning. Our proposed sampling strategy is to use tiles centered on annotated structures (UMFs, MFs, Schwann cells, and blood vessels), under-sampling them in large images, and over-sampling them in small ones by data augmentation (see section “Tile augmentation”). This sampling strategy ensures that tiles crowded by UMFs are selected more often, allowing for the network to give more emphasis on dense fiber patterns during training. This strategy alone doesn’t increase performances on the validation set and it also introduces a bias toward small fibers, because their density is inversely proportional to the area. We thus define an area-based selection score $s=A\cdot C$ where *A* is the area of the fiber and $C=\frac{4\pi A}{P^2}$ is the *circularity* of the fiber (*P* is the perimeter) to add a sampling bias toward elongated fibers, which are relatively harder to segment. The fibers are sampled using a multinomial distribution over the annotated instances, using the normalized scores as probability, and if a fiber is sampled multiple times augmentation is applied to generate unique views. This strategy gives the best *RQ*.

#### Tile augmentation

We apply two different types of tile augmentation: *center jitter* and horizontal/vertical flips. The former is applied to only tiles associated with fibers with a bounding box larger than the tile size and ten tiles are generated, each time randomly selecting a pixel inside the fiber area as the center and redefining tile boundaries. Flip augmentation is only applied to tiles selected from images where number of fibers is lower than the predefined maximum number of tiles (see section “Training parameters”) to roughly have the same number of tiles in each image.

#### Dependence on the dataset

We assess the robustness of the method on the choice of training images by performing a 5-fold cross validation on the 10 images of the training set. Two different images are selected in each fold to measure model generalization to new images; the folds are (7, 8), (11, 15), (10, 12), (9, 13), (14, 16). We follow the approach proposed by Varoquaux^32^ of comparing models on a common test set, i.e., images 17 and 18 in our case. *SQ* is $0.7912\pm 0.019$ for validation and $0.7664\pm 0.007$ for test; *RQ* is $0.8049\pm 0.07$ for validation and $0.5977\pm 0.052$ for test.

Results of this experiment show that segmentation quality scores are robust across different dataset splits with a standard deviation of less than 1% on the test data. Recognition quality scores have higher variance as small changes in the model may cause segmented fibers to split or merge making a larger relative effect on small images such as the ones in the test set. The variance of both measures is higher for the validation set, but this reflects the heterogeneity of the images in different folds and may not necessarily suggest high variance for model performance.

### Final training, inference, and post processing

The final model is trained with the entire set of images designated as *train* in Table 1 (Images 1–16) using the tile sampling strategy discussed in section “Tile sampling”. For images 1 through 6 up to 3000 tiles per image are used, whereas for images 7 through 16 up to 1500 tiles per image are used. We use the default model and the training strategy discussed in section “Training parameters”. At inference time, the image is partitioned into overlapping tiles of size $t=512$ and is processed tile by tile with a stride of $s=64$. This processing is illustrated in Fig. 5. The final segmentation map is obtained by applying majority voting to pixel-wise predictions. This method is expensive, because each pixel may receive up to 64 separate predictions, but inference times can be reduced by increasing the stride at the price of a slightly lower accuracy. For example, with $s=256$ inference times are reduced from 151 to 10 s for image 18.

Detections smaller than 50 pixels are removed as possible false positives. In the predicted masks, borders of adjacent fibers might often be found touching each other. Thus, considering pixels detected as the *border* class as part of fibers by default can lead to merging of fibers that would otherwise be properly segmented at the instance level, degrading performance. Figure 6a shows pixels assigned to border class and fibers together. If these two classes were merged an *RQ* score of only 0.155 is achieved. To avoid such situations pixels detected as the border class are assigned to background and are ignored from subsequent processing. As shown in Fig. 6b after border pixels are assigned to background most touching fibers become separated and the *RQ* score jumps to 0.876. The boundaries of individual fiber detections are dilated by up to five pixels to account for the outer edge of the fibers assigned to background. To avoid merging near contiguous fibers, an image dilation is performed iteratively one pixel at a time and is reverted for fibers that touch other fibers after each dilation. Different images may require different dilation factor, and we chose five as a good balance between under- and over-segmentation. Figure 6c shows the segmentation map after this dilation is applied. The dilation improves both $$RQ $$ and *SQ* scores.

### Validation of second-order spatial statistics of axon locations

Although validation of the segmentation output can be limited to a direct comparison of individual fiber instances, a major goal of automated segmentation of neuroanatomical TEM images is to create a comprehensive statistical description of the spatial arrangement of the axons. This critical knowledge will allow the development of biophysical models to design and build electrodes necessary to execute electrostimulation treatment^33,34^. We thus evaluate the faithfulness of the axon spatial representation produced by the deep learning model. Specifically, we compare the empirical metric of second-order spatial statistics computed from the manual and automatic segmentation maps. We use a reformulation of Ripley’s *K*-function to describe the second-order intensity statistics^35–37^.

For a stationary process with intensity $\lambda$, the value of $\lambda K(r)$ represents the number of points closer than *r* to an arbitrary other point^35^:

$$\begin{aligned} t\left( {u,r,{{\mathbf {X}}}} \right) = \sum \limits _{j = 1}^{n(x)} {{{\mathbf {1}}}\left\{ {0 < \left\| {u - {x_j}} \right\| \leqslant r} \right\} } ,\quad K\left( r \right) = \frac{{{\mathbb {E}}\left[ {t\left( {u,r,{{\mathbf {X}}}} \right) |u \in {{\mathbf {X}}}} \right] }}{\lambda } \end{aligned}$$

An estimator for an empirical *K*-function is formulated as

$$\begin{aligned} {{\hat{K}}}\left( r \right) = \frac{W}{{n\left( {1 - n} \right) }}\sum \limits _{i = 1}^n {\sum \limits _{j = 1,j \ne i}^n {{{\mathbf {1}}}\left\{ \left\| {{x_i} - {x_j}} \right\| \leqslant r \right\} } } {e_{ij}}\left( r \right) \end{aligned}$$

where *W* is the area of observation window, *n* is the number of points, $\left\| .\right\|$ is the Euclidean distance between points, and $e_{ij}$ is the edge correction weight^38^.

For some specific point processes, the explicit formula for *K* can be derived. Specifically, it can be shown that in the Poisson point process, i.e. with complete spatial randomness (CSR), the function is $K_{Pois}(r)=\pi r^2$ regardless of the intensity of the process. This result emerges from the fact that presence of a random point an any location does not impact the presence of points at other locations. On the basis of $K_{Pois}$, a transformation of *K* was proposed by Besag^37^ and named *L*-function: $L\left( r \right) = {\left( {{{K\left( r \right) } / \pi }} \right) ^{{1 / 2}}}$. The square root results in variance stabilization; therefore, the empirical variance of *L*(*r*) is approximately constant at different *r*. For ease of interpretation, we use the centered version of the *L*-function $L_{c}(r)=L(r)-r$. This operations maps the theoretical Poisson *K*-function into a straight horizontal line $L_{c,Pois}(r)=0$.

A critical property of *K*- and *L*-functions is that they are invariant to the intensity of a point process^35,39^, and to points missing at random. Consequently, the second-order statistics can be compared even if the number of points differs, as in the case of the number of axons (or axon centroids) identified by the manual segmentation process, and by our automated segmentation technique.

The complex fascicular organization of axons in the vagus nerve^34,40^, such as presence of Remak bundles^41^, suggest that one cannot a priori assume the spatial homogeneity of axon centroids. Therefore, we also evaluate an inhomogeneous *L*-function, based, analogously to the case above, on the inhomogenus *K*-function. The estimator of $K_{inh}$ is:

$$\begin{aligned} {{{{\hat{K}}}}_{inh}}\left( r \right) = \frac{1}{{{D^p}W}}\sum \limits _i {\sum \limits _{j \ne i} {\frac{{{{\mathbf {1}}}\left\{ {\left\| {{x_i} - {x_j}} \right\| \leqslant r} \right\} }}{{{{\hat{\lambda }}} \left( {{x_i}} \right) {{\hat{\lambda }}} \left( {{x_j}} \right) }}} } e\left( {{x_i},{x_j};r} \right) ,\quad {D^p} = {\left( {\frac{1}{W}\sum \limits _i {\frac{1}{{{{\hat{\lambda }}} \left( {{x_i}} \right) }}} } \right) ^p},\quad p \in \left\{ {1,2} \right\} , \end{aligned}$$

where ${{\hat{\lambda }}} (u)$ is an estimator of the density function obtained using a kernel-smoothed intensity estimator.

For the analysis, we use the open-source image processing package Fiji^42^ and spatstat R-library for spatial point pattern analysis^38^. The inhomogeneous *L*-function was evaluated using the spatstat::Linhom function. The confidence interval for CSR was estimated using 39 simulations generating random point patterns within the region of interest defined by the contour of the analyzed vagus nerve.

## Results

Multiple images are used for evaluation. Performance is quantitatively evaluated in terms of *SQ* and *RQ* scores defined in section “Training parameters” using manual segmentation maps as a reference and illustrated by overlaying automated and manual segmentation maps on original images. In all figures intersection of automated and manual segmentation maps define TP and are shown in green. Regions segmented in the automated map but not in the manual map define FP and are shown in blue. The regions segmented in the manual map but not in the automated map represent FN and are shown in red.

### Fully-automated evaluation on new cases

**Figure 7:**
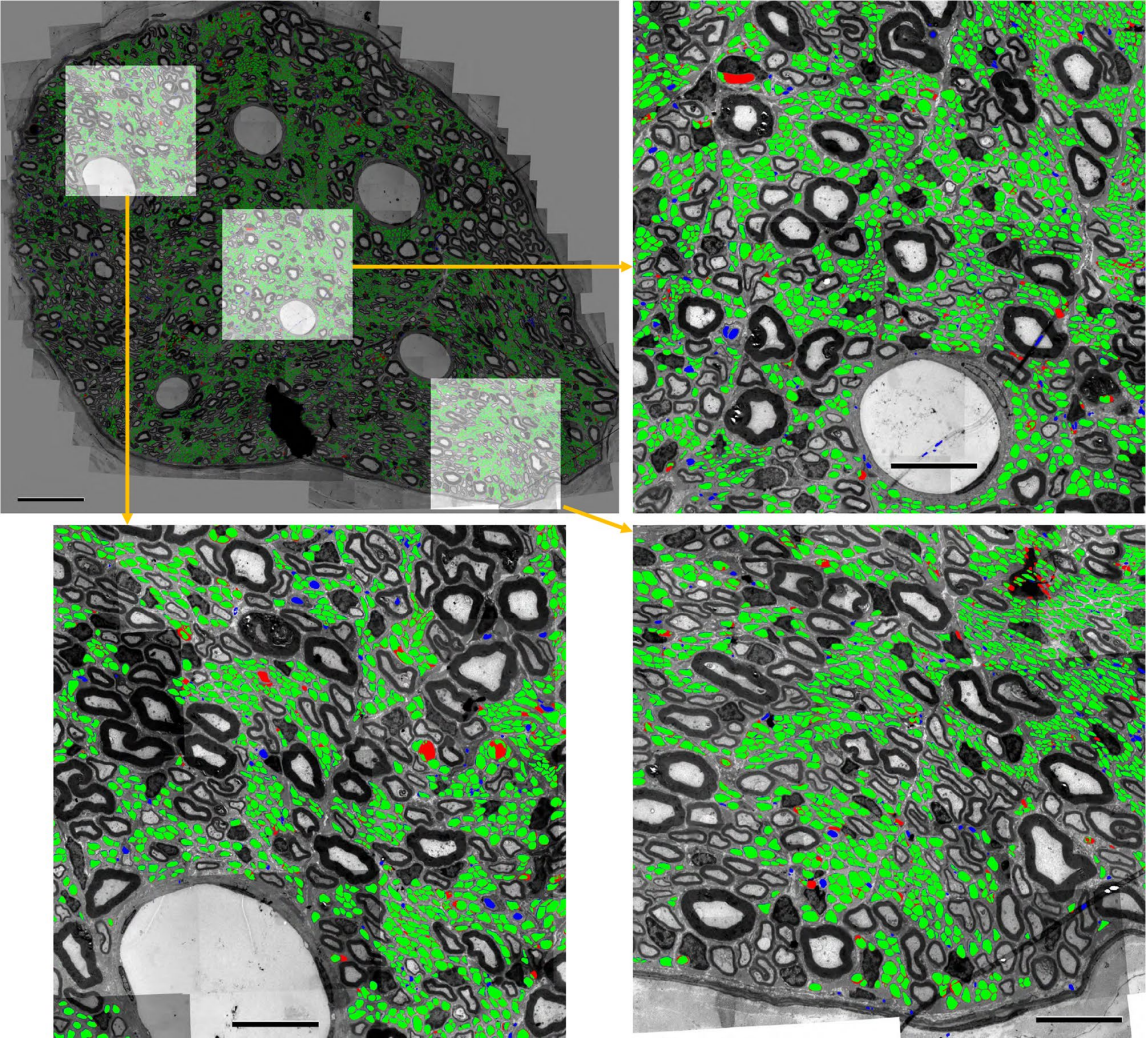
Fully automated evaluation on an image with dense regions of UMFs surrounded by myelin-rich regions. TP, FP, and FN regions are shown in green, blue, and red, respectively. Scale bars 30 $\upmu$m on the top left and 10 $\upmu$m otherwise. $SQ=0.872$ and $RQ=0.909$.

First, we evaluate the performance of the algorithm as a stand-alone, fully-automated segmentation tool on two test images: images 19 and 20 in Table 1. Image 20 is fully annotated by an expert and the image contains 12,251 UMFs. The image is best characterized by dense UMF regions surrounded by myelin-rich regions. For the entire image our algorithm achieves *SQ* and *RQ* scores of 0.872 and 0.909, respectively. Figure 7 shows the magnified views of the segmentation results from three regions in the image. An *RQ* score of 0.909 for the whole image suggest that vast majority of UMF instances are correctly detected as TPs with very few FPs and FNs. An *SQ* score of 0.872 suggest interior regions of individual UMF instances are recovered by an average *IoU* score of 87.2%. Image 19 is best characterized as a low-contrast image with relatively vague fiber borders. Only a portion of this image containing 364 fibers was manually annotated. In this sub-image our algorithm achieves an *SQ* and *RQ* scores of 0.802 and 0.707, respectively. The gray-level image is shown in Fig. 10a. Segmentation results are shown in Fig. 10b. Although the algorithm misses several UMFs in this image due to indistinct fiber borders and clumped cell patterns dominating the image, it is encouraging to see that fibers correctly detected are recovered by an average *IoU* score of 80.2%.

### Comparison with a generic cell segmentation technique

Second, we compare our results with *Cellpose*^24^, a generalist method for cell segmentation. *Cellpose*, instead of directly predicting the segmentation masks, infers the gradients of a diffusion process inside the annotated cell regions, and at test time recovers segmentation masks as basins of attraction, a process that can inherently separate adjacent cells. Image variability is handled by a style vector inspired by *StyleGAN*^43^ and computed from the image features. The method works across a wide range of image types and cell-like structures, but it still requires domain-specific training to separate different structures, e.g., unmyelinated and myelinated fibers.

We test the default cytometric model and use the built-in cell size estimation to automatically infer expected fiber diameter. For this evaluation we use image 18 in Table 1. The image is processed at full resolution in the same way our algorithm is run and inference takes 33.5 s. The result is shown in Fig. 11. The *RQ* score of 0.21 suggests CellPose misses most of the UMFs, which is also evident in Fig. 11c. CellPose cannot distinguish among different types of fibers, and thus several myelinated fibers are incorrectly detected as UMFs. The small number of UMFs correctly detected are recovered by an average *IoU* score of only 69.0%. On the same image our algorithm achieves an *RQ* score of 0.836 and recovers detected UMFs by an average *IoU* score of 80.8%.

### Validation of spatial distribution of unmyelinated fibers

**Figure 8:**
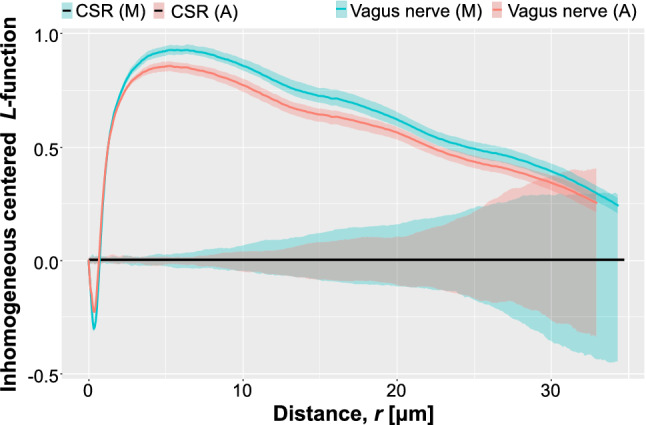
Plot of empirical *L*-functions computed for manually, and automatically segmented vagus nerve image. CSR—complete spatial randomness, A—automated segmentation, M—manual segmentation. Shaded areas shows the boundaries of 95-percentile confidence interval.

We compare the second-order statistics of the automatically and manually segmented test image (Image 20 in Table 1). Our use of *L*-function follows a similar application in neuroscience by Diggle^44^. However, we are not comparing groups of patterns, but just two patterns; therefore, we cannot use directly the method proposed by Diggle, which is based on statistics analogous to the residual sum of squares in a conventional one-way ANOVA^44^. Therefore, instead, we compute the confidence intervals of *L*-function estimations using Loh’s bootstrap method^45^, and compare whether the confidence interval bands estimated for both patterns overlap at specific values of *r* (See Fig. 8). Examination of the empirical *L*-functions shows a high level of similarity between the identified spatial patterns. Both segmentation approaches recognized the regularity of the pattern at short distances. This suggests inhibition due to the fact that axons, as physical objects, have a certain diameter; therefore, the centroids must be separated by a hardcore distance. Both methods identified the *r* value associated with the maximal difference between the number of neighbors expected for CSR and the observed patterns. Interestingly, the automated segmentation produces a larger value of *L*-function, suggesting that a greater number of closely neighboring axons were identified.

The differences in the spatial arrangements at large distances are not significant. The values of *L*-function above 0 suggest a positive association between centroids. This can be interpreted as the formation of axon clusters. Segmentation by both methods produces a pattern that demonstrates this property. At very large distances $(>30~\upmu$m), the patterns have characteristics indistinguishable from CSR.

**Figure 9:**
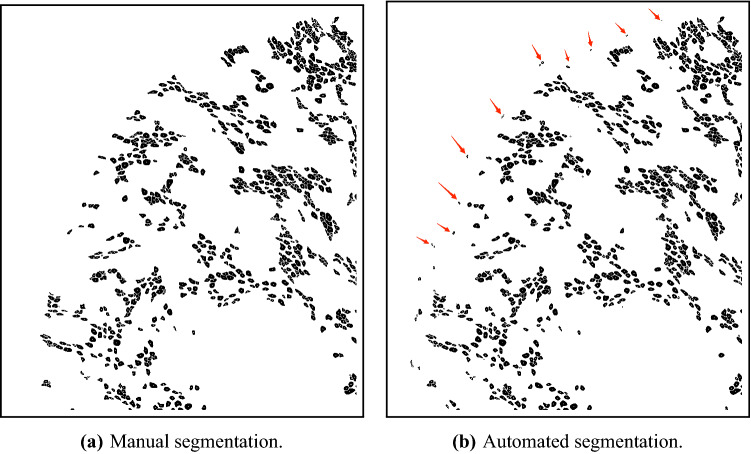
Example of axon segmentation in a cropped fragment of the test image 20. The red arrows point the spurious false positives identified at the edge of the vagus nerve. These objects located around the periphery of the region of interest affect the spatial second-order statistics of the centroids.

The *L*-function at mid-level distances shows discrepancies between the automatically extracted pattern and the manually segmented data. Although the shape of the *L*-functions remain similar, the manual segmentation identified a pattern of centroids, which is consistent with a more substantial degree of clustering, whereas the patterns determined by the the automated method slightly undervalue the degree of positive association between axons. These observations can be interpreted by visually examining the segmented test images: the false-positives (regions falsely identified as axons) close to the nerve boundary contribute to a more extensive spread (i.e., smaller level of clustering) (Fig. 9). In general: false positives will decrease the identified clustering pattern and push the *L*-function more towards the CSR. On the other hand, if they occur randomly, false negatives (missed axons) will not affect the overall characteristics of the *L*-function.

### An expert-in-the-loop evaluation

Finally, we evaluated the segmentation algorithm in a semi-automated way on an image obtained from the ventral gastric branch of a male rat (image 21 in Table 1). The binary mask generated by the segmentation algorithm is converted to an XML file where each contour in the file defines the outside border of a detected structure. An expert annotator retrieves this file in Neurolucida to refine the segmentation map as needed. The segmentation algorithm detects 4772 structures as UMFs in this image in a fully automated way. Upon visual inspection of the segmentation map by the expert, 1006 of the detected UMFs are deleted as false positives (blue regions in Fig. 12), 613 UMFs are added as false negatives (red regions in Fig. 12), and 3766 UMFs are accepted without any modifications as true positives (green regions in Fig. 12) yielding an $F_{1}$ score of 0.823 at the instance level. Any detection that requires adjustment is considered a false positive and the corresponding contour is replaced by a new manually delineated contour. Our analysis found out that the time required to refine the automated segmentation map was about 80% less than the time required to manually segment the same image from scratch (over 30 h) giving us an annotation labor savings of about 24 h in just one image.

As highlighted in Fig. 12 by the red regions, the algorithm performs quite well on small to mid-size fibers but often under-segments large and elongated ones. Increasing the number of stages to five in U-Net (see section “Training parameters”) or using multi residual ResNet^46^ did not offer much help. As large and elongated fibers are underrepresented during training we augmented any fibers that extends beyond standard tile size of 512 ten times by shifting the center of the tile uniformly each time. Although this augmentation strategy helps improve the segmentation of large and elongated fibers it does not completely eliminate the problem, most likely due to lack of diversity of these type of fibers in the training set.

## Conclusions

We presented an automated algorithm for the segmentation of unmyelinated axons by deep neural networks trained on TEM images. The method achieves high recognition scores (per-instance $F_1$ score), ranging from 0.7 to 0.9 on several test images. While the model is based on the standard U-Net architecture, our results show that careful choice of hyperparameters and other training settings play a critical role in the network’s overall performance. In particular, utilizing training tiles centered on fibers and selectively augmenting tiles based on fiber characteristics significantly improved segmentation accuracy. The introduction of a border class ensured the correct separation of individual fiber instances.

High accuracy achieved for instance-segmentation enables downstream processing. It paves the way for statistical analyses of spatial distributions of fibers in healthy and pathological tissues and the potential discovery of biomarkers and surrogate endpoints of neurological diseases. In the semi-automated mode, the algorithm cuts manual annotation time by 80%. Since TEM images may contain tens of thousands of fibers, this translates into saving hundreds of hours of researchers’ time for each image.

The implemented system operates robustly irrespective of nerve type, location, and sex of the donor-animal. However, its performance on large and elongated (elliptical) axon cross sections is suboptimal. It may also perform poorly on very low-contrast images or images with resolutions significantly outside the training range. The model has been trained only on images representing important peripheral nerves. It will likely require re-training or fine-tuning when used on images obtained from different species (e.g., humans, primates) or from the central nervous system.

Future work will consider nested U-Net architectures^47,48^ to accommodate fibers with arbitrary shapes and sizes better. We also plan to explore non-parametric Bayesian extensions^49,50^ to achieve open-set instance segmentation. The segmentation scope will be expanded with vagus nerve images from other species, particularly primates and humans. To obtain a complete map of the nerve fibers our specialized model will be extended to myelinated fibers and Schwann cells. New data augmentation techniques that can handle images with different resolutions will also be explored and implemented. Finally, we will research the concept of incorporating the constraints imposed by the second-order statistics (spatial arrangement of axons) directly into the model. Although some previous work on learning from point patterns exists, the context of this analysis was different^51^. Recent developments in spatially aware deep learning in biological applications suggest that there is a distinct value in performing classification on points to capture spatial relationships^52,53^. In our setting, it would mean an additional set of layers dedicated to recognizing the spatial arrangement of axons and other biological structures present in nerve cross sections.

**Figure 10:**
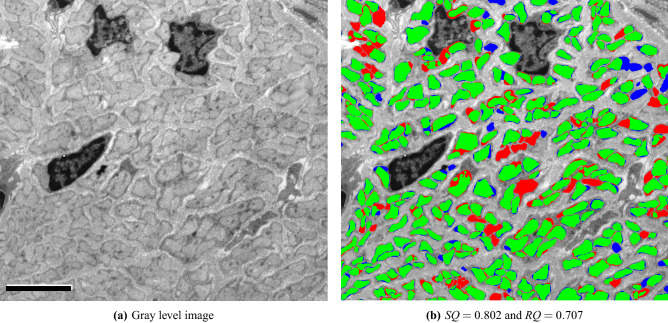
Fully automated evaluation on a low-contrast image with indistinct fiber borders and clumped patterns. TP, FP, and FN regions are shown in green, blue, and red, respectively. Scale bar: 4 $\upmu$m.

**Figure 11:**
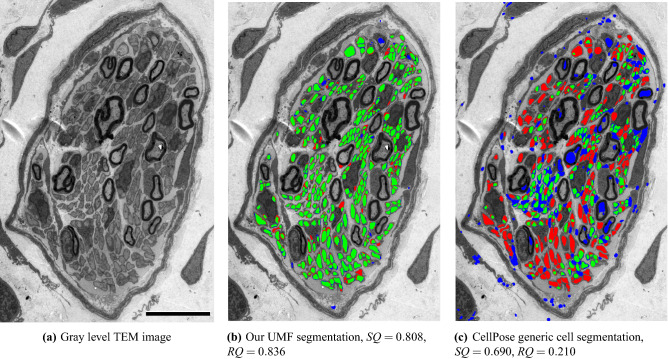
Comparing proposed model trained on TEM images annotated for UMFs against CellPose, a generic cell segmentation model trained on a wide range of cell images. TP, FP, and FN regions are shown in green, blue, and red, respectively. Scale bar: 10 $\upmu$m.

**Figure 12:**
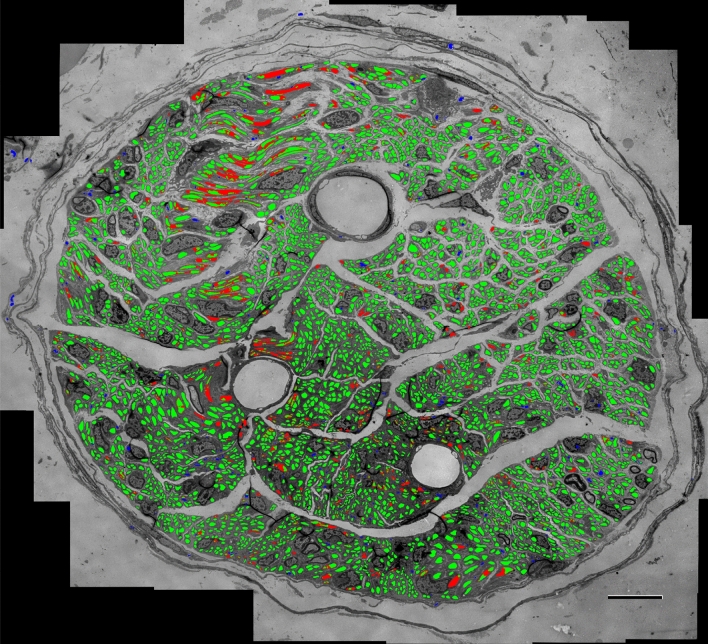
Evaluation of the segmentation algorithm by an expert on an image of size $9935 \times 8870$ obtained from the ventral gastric branch of a male rat. A total of 4772 structures were detected as UMFs. Expert deletes 1006 of them as false positives (blue), adds 613 new ones as false negatives (red) while accepting 3766 structures unmodified as true positives (green). Automated segmentation achieves an $F_1$ score of 0.823 and about 80% annotation labor savings. Scale bar: 10 $\upmu$m.

## Data Availability

All training, testing, and evaluation scripts are hosted on a GitHub repository at this address https://github.com/Banus/umf_unet. Data associated with this study^54^, were collected as part of the Stimulating Peripheral Activity to Relieve Conditions (SPARC) program and are available through the SPARC Portal (RRID: SCR_017041) under a CC-BY 4.0 license.
